# Supplementary material for: The causal relationship between the human gut microbiota and pyogenic arthritis: a Mendelian randomization study
Source: Front Cell Infect Microbiol. 2024 Nov 26;14:1452480. doi: 10.3389/fcimb.2024.1452480 (PMC11629706; doi:10.3389/fcimb.2024.1452480)
Supplement: Supplementary file 1 [file DataSheet1.zip › mendelian test/ebi-a-GCST90027611.csv_forest.pdf]

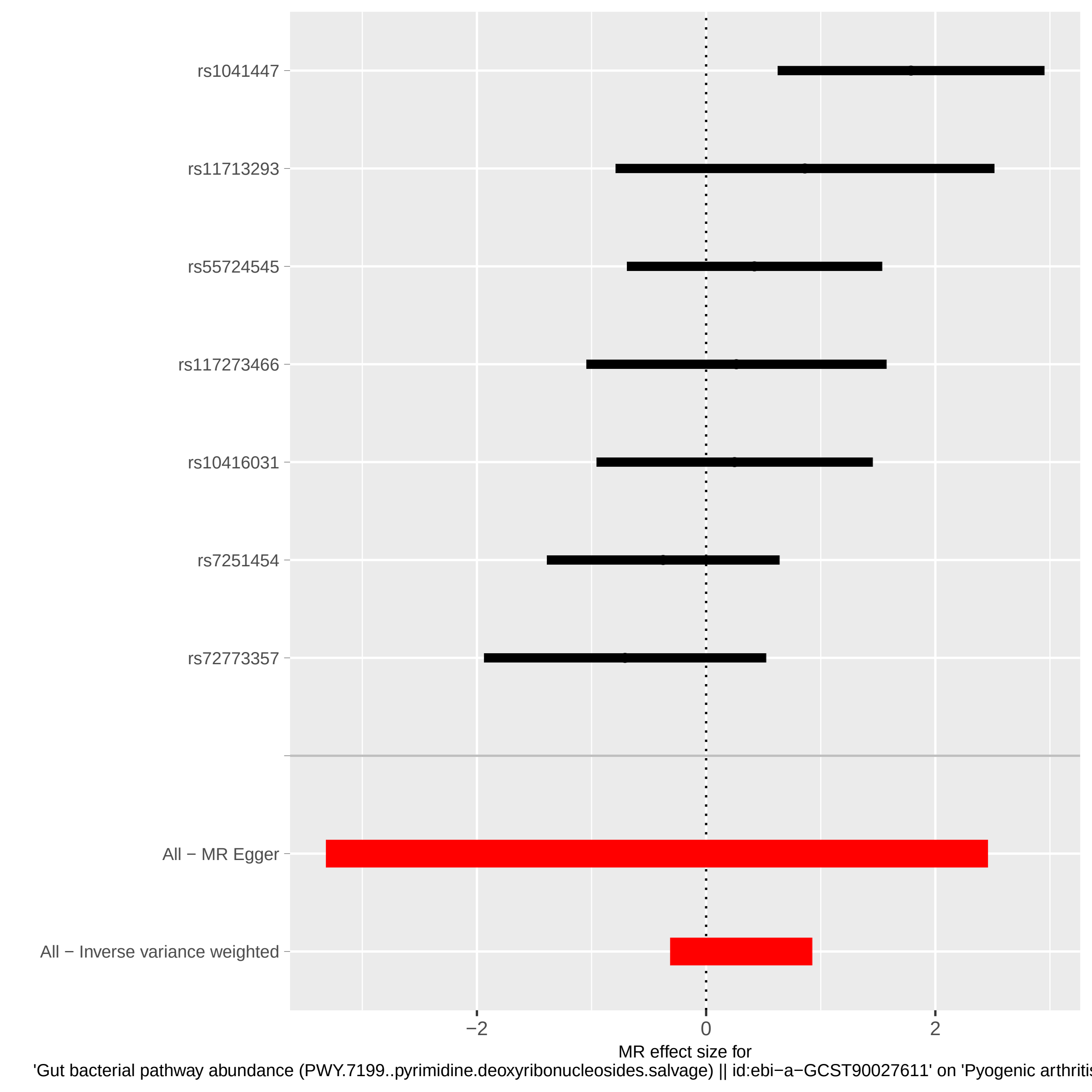
rs1041447
rs11713293
rs55724545
rs117273466
rs10416031
rs7251454
rs72773357
All − MR Egger
All − Inverse variance weighted
−2
0
2
MR effect size for
'Gut bacterial pathway abundance (PWY.7199..pyrimidine.deoxyribonucleosides.salvage) || id:ebi−a−GCST90027611' on 'Pyogenic arthritis
